# Building a second-opinion tool for classical polygraph

**DOI:** 10.1038/s41598-023-31775-6

**Published:** 2023-04-17

**Authors:** Dmitri Asonov, Maksim Krylov, Vladimir Omelyusik, Anastasiya Ryabikina, Evgeny Litvinov, Maksim Mitrofanov, Maksim Mikhailov, Albert Efimov

**Affiliations:** 1 Sber Innovation and Research, Sberbank of Russia, Moscow, 117997 Russian Federation; 2 Internal Security Department, Sberbank of Russia, Moscow, 117997 Russian Federation; 3 University of Science and Technology MISIS, Moscow, 119049 Russian Federation

**Keywords:** Psychology, Computer science

## Abstract

Classical polygraph screenings are routinely used by critical businesses such as banking, law enforcement agencies, and federal governments. A major concern of scientific communities is that screenings are prone to errors. However, screening errors are not only due to the method, but also due to human (polygraph examiner) error. Here we show application of machine learning (ML) to detect examiner errors. From an ML perspective, we trained an error detection model in the absence of labeled errors. From a practical perspective, we devised and tested successfully a second-opinion tool to find human errors in examiners’ conclusions, thus reducing subjectivity of polygraph screenings. We report novel features that uplift the model’s accuracy, and experimental results on whether people lie differently on different topics. We anticipate our results to be a step towards rethinking classical polygraph practices.

## Introduction

Safety of clients’ money and data (e.g. transactions) is at the heart of banking culture and reputation. As one of the instruments to safeguard clients, banks use polygraph screenings (PS). These are performed when hiring candidates to prevent the hiring of untrustworthy people. To detect an infringement early, employees with sensitive roles are screened regularly. The PS topics include drug abuse, gambling addiction, insider trading, disclosure of confidential information, bribery, corruption, and misappropriation and fraud (sample screening questions are in Suppl. Table 2). The finance industry is not alone in applying PS; other examples being critical sectors such as aviation, manufacturing companies, and federal law enforcement agencies throughout the world^1,2^.

The classical polygraph is a device that records cardiovascular activity (such as heart rate), thoracic and abdominal respirations, galvanic skin response (a.k.a. electrodermal activity, or EDA), and tremor. An examiner asks questions of, and accepts «*yes*» or «*no*» answers from, the person being screened (examinee). There are many good overviews of classical polygraph and questioning methods^3–5^.

Unorthodox lie detection studies analyze video and audio^6^ (including facial expressions^7,8^, pupil reaction^9^, and delays between question and answer^10^^)^, electromyography (EMG)^11^, electroencephalogram (EEG)^12^, magnetic resonance tomography (MRT)^13,14^, or writing pattern (keystroke dynamics)^15^ in addition to or instead of classical polygraph data. Some of these studies even get a chance to pilot in the new fields, such as the iBorderCtrl lie detector pilot in EU airports^16,17^ or the VeriPol deception detection pilot by Spanish police on written insurance claims^18,19^. Yet, in the traditional fields, we are unaware of any cases where classical polygraphs are substituted with unorthodox systems. Classical polygraph remains the instrument of choice in the traditional areas, such as hiring screening, and criminal and internal investigations.

Polygraph has a long history of drawing criticism from psychology^20,21^ and law scientists^22^, as well as from the public and state^1,23^. A major concern is that this method does not detect lie and truth reliably. And yet, “*paradoxically, although Congress expressed deep concerns about the efficacy of the technology, the EPPA permits the use of lie detectors in circumstances in which the accuracy of the results is of paramount importance: national defense, security, and legitimate ongoing investigations*”^22^.

Critical related work provides many arguments for why polygraph screening may fail at detecting a lie or mark a truthful answer as a lie. For example: “*Polygraph tests do not assess deceptiveness, but rather are situations designed to elicit and assess fear*”^24^. A truthful junior manager may fear being called a corruptor more than a coldblooded, corrupted senior manager fears being caught lying by a polygraph examiner. Another example of constructive critique is a grounded call for standardization of polygraph screening procedures and examiner education^25^. Of all concerns, in this paper we tackle only one: the need for quality assessment (QA) of examiner work. Examiner errors happen, for example, when an examiner is inexperienced, exhausted or distracted, or biased^26^.

A simple QA solution exists: always have another examiner review the screening and confirm or disprove the conclusions of the original examiner^27^. To QA a polygraph examiner report, another examiner needs to review the recording of the screening, including the polygram (a graphical representation of recorded sensor data coupled with the examiner’s questions and the examinee’s answers), sometimes audio and video recording, and to compare his conclusions with the original report. In our experience, QA takes at least half the time it took to perform the screening. An average screening takes at least two hours. Thus, QAs are costly in terms of both time and money. For this reason, and to the best of our knowledge, industrial internal security departments QA screenings infrequently or not at all. We also note that having other examiners to QA all screenings is not a bulletproof solution. Some examiners mistakes come not from the examiner’s bias or fatigue, but from the fact that the case is hard. In hard cases, the second examiner may make just the same mistake the original examiner did.

The overview of our experimental framework is as follows. Our main approach is to train a binary classifier model and apply it to each of the real 2094 screenings in our possession to see if the model score contradicts the examiner conclusion. To avoid applying the model to screenings that it saw during the training, we use standard stratified fivefold validation. Here we hypothesize that the model will not train to make the errors that human examiners do because the share of examiner errors is minor. We also decide to deviate from related work by not implementing polygraph examiner rules as features. We do this to avoid our model being trapped in the same way that human examiners are trapped, when some rules are disputable and have exceptions. Our secondary experimental goals are as follows:

- Ideate and test novel features that would uplift the AUC of the models. In particular, consider features of novel (not physiological) nature, such as job description and magnetic storms.
- Build models for each screening topic individually, to see if this uplifts the quality and if the AUCs of models differ from topic to topic.

The primary success benchmark of our experiments is the success in finding real examiner errors in the screenings marked by the models we trained. We implement this primary benchmark by handing the screenings flagged by the model as examiners’ errors to the human examiners for verification. The secondary benchmark that we used during the modelling process is the AUC of the models. This secondary benchmark reflects only indirectly how good the models are at catching the examiners’ errors because AUC is calculated on noisy targets containing these unknown errors. The third benchmark is the best AUC of most related work (0.85 by Slavcovic^4^). This AUC can be considered an upper bound, because it is obtained on criminal investigation polygraph data, known to have much more predictive power than job screenings^3^.

Here we report devising and testing in the field an ML tool to QA the examiner reports for PS performed on classical polygraph. A small number of reports, marked suspicious by this tool, will be handed to another examiner for QA. Such a tool would allow for semi-automatic double-checking of all new and historical reports, without hiring additional examiners. An additional advantage of such a tool is that if examiners are sure all their work will be QA-ed, they will make decisions more carefully.

Our results neither justify nor solidify the practice of classical polygraph screenings. Rather, we consider our results as a temporary and partial patch that helps to eliminate a specific type of error of this method, until better methods are devised and put into practice. More broadly, we believe we make a step towards rethinking classical polygraph practices.

Below we describe the steps, from a basic model to a validation of the final model, that succeeded at exposing real examiner errors in historical field screenings.

## Results

### Basic second-opinion model on examiner conclusions

We built a basic second-opinion model by training a model on the historical data of 2094 field polygraph screening recordings (PSRs) including Deception Indicated (DI) attributes set by the examiners who conducted the screenings. The intended use is to raise a red flag whenever an examiner conclusion contradicts the conclusion inferenced by the model.

**Figure 1:**
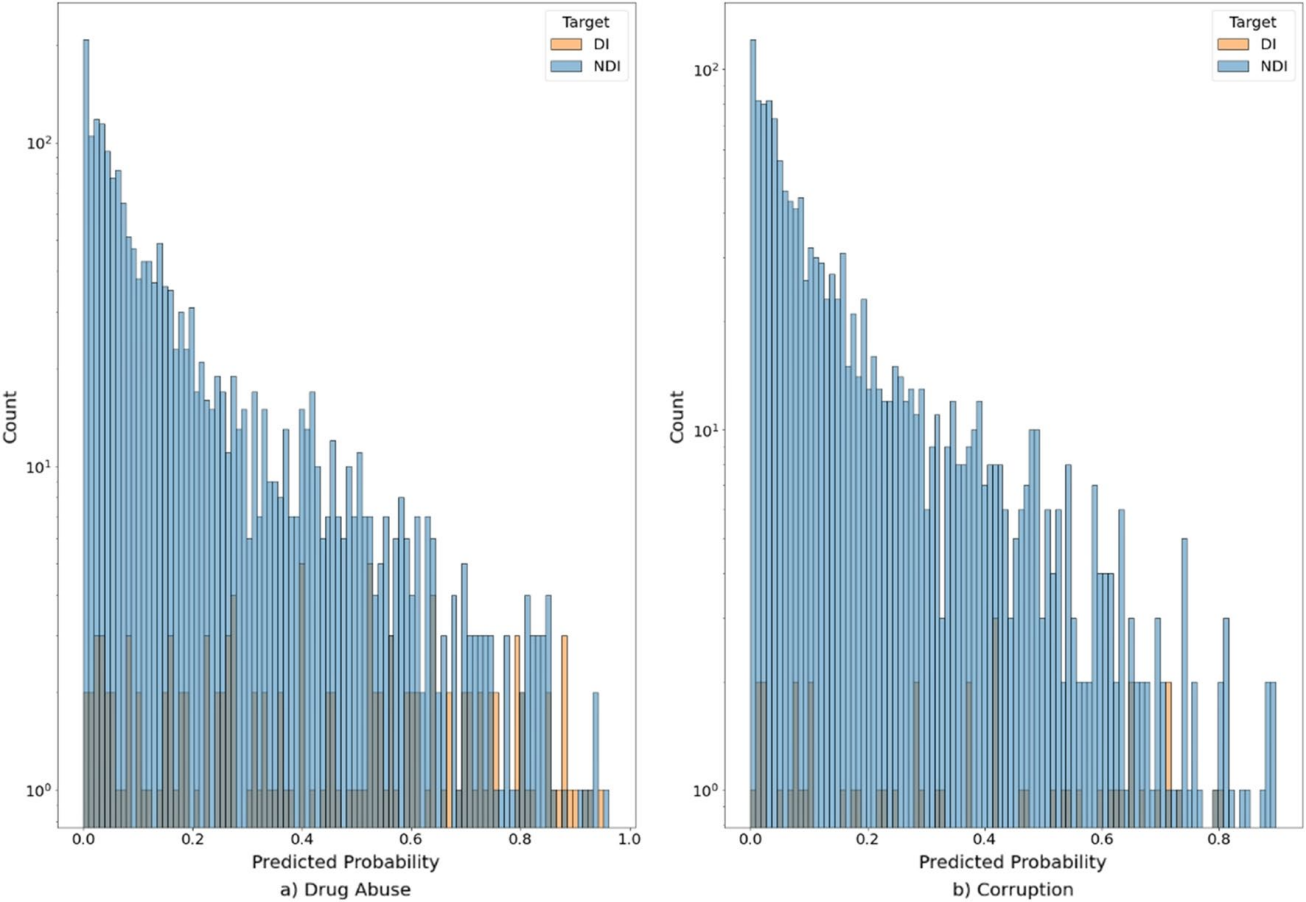
Distribution of basic model scores for topics: (**a**) “Drug abuse”; (**b**) “Corruption”.

We present the quality metrics of the basic model in Table 1a, in column «all topics». Our major quality metrics are ROC AUC, and TPR for an FPR at 0.05. We note that we are forced to use these indirect quality metrics, because they measure how the model mimics the conclusions of the examiners. In fact, counter-intuitively, and contrary to the goals of related work, we do not want a perfect model predicting examiner conclusions in up to 100% screenings, because then we would flag no candidates for examiner errors. In practice, we are interested in validating that the model detects erroneous examiner conclusions, of which at this stage we had absolutely no knowledge. Tables 2 and 3 present the importance of each of the features based on 10 raw polygraph signals and age and sex of the examinees; the details of second-level feature construction are described in the section on “Methods”. Figure 1 depicts where the model and an examiner’s conclusions agree and disagree, depending on the model score.

**Table 1:** Three models inference each of seven screening topics, measured using stratified K-fold cross-validation.

	All topics	Drug abuse	Corruption	Conf. info leak	Debt	Unrep. income	Crime history	IRD violation
ROC AUC (Std.)	a. 0.75 (0.02)	0.79 (0.02)	0.71 (0.04)	0.73 (0.05)	0.64 (0.03)	0.81 (0.03)	0.83 (0.06)	0.69 (0.03)
	b. 0.80 (0.01)	0.85 (0.02)	0.81 (0.03)	0.77 (0.05)	0.74 (0.07)	0.80 (0.09)	0.90 (0.03)	0.7 (0.03)
	c. 0.79 (0.01)	0.84 (0.02)	0.82 (0.01)	0.76 (0.05)	0.69 (0.06)	0.80 (0.02)	0.90 (0.03)	0.69 (0.04)
Recall (Std.)	a. 0.28 (0.01)	0.34 (0.01)	0.27 (0.10)	0.30 (0.08)	0.12 (0.06)	0.26 (0.09)	0.16 (0.08)	0.2 (0.07)
	b. 0.30 (0.02)	0.42 (0.03)	0.25 (0.09)	0.15 (0.07)	0.20 (0.05)	0.27 (0.09)	0.54 (0.19)	0.13 (0.05)
	c. 0.29 (0.02)	0.40 (0.03)	0.22 (0.05)	0.24 (0.07)	0.15 (0.04)	0.37 (0.06)	0.47 (0.14)	0.09 (0.05)
Precision (Std.)	a. 0.14 (0.01)	0.32 (0.06)	0.14 (0.04)	0.10 (0.03)	0.04 (0.02)	0.08 (0.02)	0.05 (0.03)	0.10 (0.04)
	b. 0.19 (0.02)	0.45 (0.08)	0.17 (0.06)	0.06 (0.03)	0.12 (0.03)	0.10 (0.04)	0.12 (0.04)	0.09 (0.04)
	c. 0.18 (0.02)	0.41 (0.06)	0.16 (0.02)	0.11 (0.04)	0.08 (0.03)	0.12 (0.02)	0.11 (0.03)	0.06 (0.03)
F1_score (Std.)	a. 0.19 (0.01)	0.32 (0.04)	0.18 (0.06)	0.14 (0.04)	0.06 (0.03)	0.12 (0.03)	0.08 (0.04)	0.14 (0.05)
	b. 0.23 (0.02)	0.43 (0.05)	0.2 (0.07)	0.09 (0.04)	0.14 (0.04)	0.14 (0.05)	0.18 (0.06)	0.1 (0.04)
	c. 0.22 (0.02)	0.40 (0.04)	.18 (0.03)	0.15 (0.05)	0.10 (0.03)	0.18 (0.02)	0.16 (0.04)	0.07 (0.04)
Accuracy (Std.)	a. 0.92 (0.00)	0.90 (0.01)	0.93 (0.00)	0.94 (0.00)	0.93 (0.01)	0.94 (0.01)	0.93 (0.01)	0.92 (0.01)
	b. 0.93 (0.01)	0.92 (0.01)	0.94 (0.00)	0.94 (0.01)	0.94 (0.01)	0.94 (0.01)	0.93 (0.01)	0.91 (0.01)
	c. 0.93 (0.01)	0.92 (0.01)	0.94 (0.01)	0.94 (0.01)	0.94 (0.01)	0.94 (0.01)	0.93 (0.01)	0.92 (0.01)
TNR (Std.)	a. 0.95 (0.00)	0.94 (0.01)	0.95 (0.00)	0.95 (0.00)	0.95 (0.01)	0.95 (0.01)	0.94 (0.01)	0.94 (0.01)
	b. 0.95 (0.01)	0.96 (0.01)	0.96 (0.00)	0.95 (0.01)	0.96 (0.01)	0.95 (0.01)	0.94 (0.01)	094 (0.01)
	c. 0.95 (0.01)	0.96 (0.01)	0.96 (0.00)	0.96 (0.01)	0.96 (0.01)	0.96 (0.01)	0.94 (0.01)	0.95 (0.01)
FPR (Std.)	a. 0.05 (0.00)	0.06 (0.01)	0.05 (0.00)	0.05 (0.00)	0.05 (0.01)	0.05 (0.01)	0.06 (0.01)	0.06 (0.01)
	b. 0.05 (0.01)	0.04 (0.01)	0.04 (0.00)	0.05 (0.01)	0.04 (0.01)	0.05 (0.01)	0.06 (0.01)	0.06 (0.01)
	c. 0.05 (0.01)	0.04 (0.01)	0.04 (0.00)	0.04 (0.01)	0.04 (0.01)	0.04 (0.01)	0.06 (0.01)	0.05 (0.01)
Number of DI	261	137	48	36	32	24	24	47

(a) Basic model (features based on 10 raw polygraph signals, age and sex of the examinees). (b) Basic model with alternative data. (c) Basic model with job position data.

**Table 2:** Feature importance of the first-level basic model.

	Feature	Importance
1	ABS_BLOOD_VOLUME_min_min_mean	0.89
2	PLE_mean_diff_mean	0.83
3	ABS_BLOOD_VOLUME_min_mean_mean	0.75
4	ABS_BLOOD_VOLUME_max_max_mean	0.72
5	ABS_BLOOD_VOLUME_max_max_max	0.70
6	OPTIONAL_std_max_max	0.68
7	ABS_BLOOD_VOLUME_min_mean_max	0.66
8	ABS_BLOOD_VOLUME_mean_mean_mean	0.65
9	ABS_BLOOD_VOLUME_mean_max_mean	0.61
10	EDA_std_mean_max	0.57
11	ABS_BLOOD_VOLUME_mean_max_max	0.56
12	OPTIONAL_std_mean_max	0.54
13	EDA_mean_diff_max	0.52
14	OPTIONAL_std_mean_mean	0.50
15	ABDOMINAL_RESP_mean_diff_max	0.49
16	OPTIONAL_std_max_mean	0.48
17	TREMOR_mean_max_max	0.47
18	TREMOR_min_mean_mean	0.47
19	TONIC_EDA_amplitude_mean_max	0.47
20	TONIC_EDA_std_mean_max	0.46

**Table 3:** Feature importance of the second-level basic model.

1	Feature	Importance
2	subject_age	28.56
3	pred_proba_min	19.53
4	pred_proba_max	17.53
5	pred_proba_mean	16.50
6	pred_proba_diff	8.96
7	subject_sex	8.93

We also decided to measure the quality of the model applied to each of seven screening topics (Table 1a). To the best of our knowledge, we are the first to report the model quality on separate topics within a standard employee screening, and this approach will help us as shown below.

### Using alternative data to improve the model quality

Measuring the performance of a model involves spending approx. 100 examiner hours, because it involves a real examiner QA-ing thoroughly dozens of PSRs flagged by the model. In case of failure, i.e. in the case of finding no examiner errors in the flagged screenings, no second chance would be given to waste another 100 h of the limited resource. We tried to maximize our one chance by doing our best to increase the quality of the basic model before flagging suspicious conclusions for manual QA.

We hypothesized that information about geomagnetic storms on Earth^28^ and weather conditions in the city on the date of the screening may help the model to predict. The intuition behind this assumption is that, during storms and under different weather conditions, humans might behave slightly differently, resulting in slightly different raw physiological measurements or the sensors might provide slightly shifted measurements, or both.

We also collected examiner ID, hoping that these data may be of help to the model, because different examiners might provoke slightly different physiological reactions in examinees, or the polygraph devices assigned to each examiner might have slightly different signal measurement deviations. We also collected roles (e.g. job positions) of the examinees, because people of different education and training may tell the truth and lie differently.

Table 1b presents a basic model re-trained with these alternative data and Table 4 shows the importance of these features (full description of all features is in Suppl. Table 6). The uplift each data source is providing to the model based only on physiological signals is shown in Table 5.

**Table 4:** Feature importance of the second-level basic model with alt data.

	Feature	Importance
1	subject_age	8.52
2	pred_proba_min	6.98
3	pred_proba_max	6.64
4	examiner_id	6.59
5	pred_proba_mean	6.26
6	Pressure	5.83
7	current_position	5.55
8	Wind	5.25
9	Dew Point	4.83
10	accepted_position	4.75
11	Humidity	4.62
12	pred_proba_diff	4.24
13	current_department	4.18
14	Condition	4.17
15	Temperature	4.00
16	Time	3.63
17	accepted_department	3.08
18	Wind speed	3.04
19	subject_sex	2.41
20	subject_type	2.31

**Table 5:** Uplifts of AUC from each alternative data source for entire dataset, and for each topic.

	Without alt. data	Examinee age	Examinee sex	Job position	Weather	Geomagn. storms	Examiner id
Overall	0.75 (0.02)	+ 4%	+ 2%	** + 8%**	+ 4%	0%	+ 3%
Drug abuse	0.79 (0.02)	** + 6%**	+ 3%	+ **11%**	** + 9**%	0%	+ **5%**
Corruption	0.71 (0.04)	− 2%	+ 1%	+ **7%**	− 3%	+ 1%	** + 5%**
Conf. info leak	0.73 (0.05)	+ **5%**	+ 4%	+ **7%**	+ 4%	+ 3%	+ 1%
Debt	0.64 (0.03)	0%	− 2%	+ **6%**	+ 3%	− 2%	+ 1%
Unrep. income	0.81 (0.03)	+ **6%**	+ **6%**	+ **8%**	+ 3%	− 1%	** + 6%**
Criminal history	0.83 (0.06)	+ 3%	+ **5%**	+ **11%**	+ **6%**	+ 4%	+ 3%
IRD violation	0.69 (0.03)	+ **5%**	− 1%	+ 2%	0%	0%	+ 3%

Significant values are in bold.

All alternative data types uplifted the quality of the model; however, we decided to keep only age, sex and job roles for production (Table 1c). Weather showed anomalously high uplift and importance, and we feared that this is because, for technical reasons, our dataset is highly unbalanced by the percentage of DI labels per city. To exclude city bias, we cut the dataset to one city but weather still was high in feature importance. Thus, we believe weather is significant alternative data. However, on the full dataset, weather could leak city information, and the model could get the city bias from the unbalanced dataset. While examiner ID provided a moderate uplift, the nature of this feature requires further investigation before relying on it in production. For example, if it is not the examiner ID but the examiner’s polygraph device ID that helps inferencing, then we will have wrong scoring when an examiner changes his device.

### A model built for one topic performs marginally better

Here we investigate if training a separate model for each topic will result in even better quality as compared to the basic model with job position. We had DI labels enough for training for one topic only, i.e. for drug abuse (137 DI labels). Table 6 shows that we gain a + 2% ROC AUC (6% relative uplift) if we train a model for the drug abuse topic only. This allows us to speculate that people may lie differently on different topics, and thus separating the topics makes it easier for the model to learn and to inference. More data and research are needed to confirm this hypothesis.

**Table 6:** A model built for “Drug abuse” topic inferences each of seven screening topics, measured using stratified K-fold cross-validation.

	All topics	Drug abuse	Corruption	Conf. info leak	Debt	Unrep. income	Criminal history	IRD violation
ROC AUC	0.80 (0.01) ∆ = + 1%	0.86 (0.02) ∆ = + 2%	0.80 (0.04) ∆ = − 2%	0.80 (0.04) ∆ = + 4%	0.72 (0.06) ∆ = + 3%	0.81 (0.04) **∆ = ** + 1%	0.90 (0.03) ∆ = 0%	0.69 (0.03) **∆ = **0%
Recall	0.25 (0.02) ∆ = − 4%	0.31 (0.02) ∆ = − **9%**	0.19 (0.07) ∆ = − 3%	0.25 (0.09) **∆ = ** + 1%	0.17 (0.05) ∆ = + 2%	0.18 (0.09) **∆ = **− **19%**	0.46 (0.16) ∆ = − 1%	0.17 (0.03) ∆ = ** + 8%**
Precision	0.16 (0.02) ∆ = − 2%	0.37 (0.04) **∆ = **− 4%	0.17 (0.08) **∆ = ** + 1%	0.11 (0.05) ∆ = 0%	0.10 (0.04) **∆ = ** + 2%	0.06 (0.03) **∆ = **− **6%**	0.09 (0.02) **∆ = **− 2%	0.08 (0.02) **∆ = ** + 2%
F1_score	0.22 (0.02) ∆ = − 3%	0.33 (0.04) ∆ = − **7%**	0.17 (0.07) ∆ = − 1%	0.15 (0.06) ∆ = 0%	0.12 (0.04) ∆ = + 2%	0.09 (0.04) ∆ = − **9%**	0.14 (0.04) ∆ = − 2%	0.11 (0.02) ∆ = + 3%
Accuracy	0.93 (0.01) ∆ = 0%	0.91 (0.01) ∆ = − 1%	0.93 (0.01) ∆ = − 1%	0.95 (0.00) ∆ = + 1%	0.94 (0.01) ∆ = 0%	0.95 (0.01) ∆ = + 1%	0.93 (0.01) ∆ = 0%	0.91 (0.01) ∆ = − 1%
TNR	0.95 (0.01) ∆ = 0%	0.96 (0.01) ∆ = 0%	0.96 (0.01) ∆ = 0%	0.96 (0.01) ∆ = 0%	0.96 (0.01) ∆ = 0%	0.96 (0.01) ∆ = 0%	0.94 (0.01) ∆ = 0%	0.93 (0.01) ∆ = − 2%
FPR	0.05 (0.01) ∆ = 0%	0.04 (0.01) ∆ = 0%	0.04 (0.01) ∆ = 0%	0.04 (0.01) ∆ = 0%	0.04 (0.01) ∆ = 0%	0.04 (0.01) ∆ = 0%	0.06 (0.01) ∆ = 0%	0.07 (0.01) ∆ = + 2%
Number of DI	261	137	48	36	32	24	24	47

Significant values are in bold.

### A model built on one topic can handle other topics with varying quality

We applied the Drug Abuse model from the previous paragraph to inferencing the other six topics (Table 6). Compared to the universal model, the performance of the Drug Abuse model varies from topic to topic. We conclude that a model trained on one topic can handle other topics, albeit with insignificant quality degradation for some topics.

### Vague questions are hard not only for people and examiners, but for models too

In Table 1 we observed that the basic model performs on some topics (such as drug abuse and criminal history) significantly better than on other topics (such as unreported income or IRD violation). This observation is in line with a long-standing issue in the screenings: people just cannot confidently answer questions when they are not sure about the answer. At the bank, we have hundreds of IRDs, dozens of pages each, not to mention the versioning, so some people are not sure if they never violated a single IRD. Similarly with unreported income: some people start asking themselves questions like «*if I got cash from a relative, is this an income?*», etc. As with any common knowledge without quantitative proof, there were heated debates whether topics like «IRD violation» are effective or need to be more specific. Our finding helped to end this never-ending discussion in our organization.

We also could use this observation to improve the quality of the basic model. If we found a couple of topics that confuse people, the basic model must be confused training on these. We tried to remove these confusing labels from the trainset all together. However, and counter-intuitively, in Table 7 we can see that this idea did not improve the quality of the model significantly, and the quality of the inferencing for confusing topics dropped or did not change. We presume we did not observe a significant positive effect because the share of confusing topic DI labels in the trainset is insignificant.

**Table 7:** Basic model without “IRD violation” and “Unreported income” topics in the train set.

	All topics	Drug abuse	Corruption	Conf. info leak	Debt	Unrep. income	Criminal history	IRD violation
ROC AUC	0.81 (0.01) ∆ = + 2%	0.87 (0.01) ∆ = + 3%	0.82 (0.03) ∆ = 0%	0.76 (0.04) ∆ = 0%	0.74 (0.06) ∆ = ** + 5%**	0.81 (0.03) ∆ = + 1%	0.93 (0.02) ∆ = + 3%	0.67 (0.03) ∆ = + 2%
Recall	0.34 (0.02) ∆ = + **5%**	0.43 (0.03) ∆ = + 3%	0.29 (0.06) ∆ = ** + 7%**	0.27 (0.09) ∆ = + 3%	0.29 (0.04) ∆ = + **14%**	0.25 (0.11) ∆ = − **12%**	0.6 (0.05) ∆ = + **13%**	0.21 (0.04) ∆ = + **12%**
Precision	0.18 (0.02) ∆ = + 1%	0.42 (0.07) ∆ = + 1%	0.2 (0.04) ∆ = + 4%	0.09 (0.03) ∆ = − 2%	0.14 (0.03) **∆ = + 6%**	0.06 (0.03) **∆ = **− **6%**	0.13 (0.06) ∆ = + 2%	0.09 (0.02) ∆ = + 3%
F1_score	0.23 (0.02) ∆ = ** + 5%**	0.42 (0.05) ∆ = + 2%	0.23 (0.04) ∆ = + **5%**	0.13 (0.04) ∆ = − 2%	0.18 (0.03) ∆** = + 8%**	0.09 (0.04) ∆ = − **9%**	0.20 (0.06) ∆ = + 4%	0.12 (0.03) ∆ = + **5%**
Accuracy	0.93 (0.01) ∆ = 0%	0.92 (0.01) ∆ = 0%	0.94 (0.00) ∆ = 0%	0.93 (0.00) ∆ = − 1%	0.94 (0.01) ∆ = 0%	0.94 (0.00) ∆ = 0%	0.93 (0.01) ∆ = 0%	0.90 (0.01) ∆ = − 2%
TNR	0.95 (0.01) ∆ = 0%	0.95 (0.01) ∆ = − 1%	0.96 (0.00) ∆ = 0%	0.95 (0.01) ∆ = − 1%	0.95 (0.01) ∆ = − 1%	0.95 (0.01) ∆ = − 1%	0.94 (0.01) ∆ = 0%	0.92 (0.01) ∆ = − 3%
FPR	0.05 (0.01) ∆ = 0%	0.05 (0.01) ∆ = + 1%	0.04 (0.00) ∆ = 0%	0.05 (0.01) ∆ = + 1%	0.05 (0.01) ∆ = + 1%	0.05 (0.01) ∆ = + 1%	0.06 (0.01) ∆ = 0%	0.08 (0.01) ∆ = + 3%
Number of DI	261	137	48	36	32	24	24	47

Significant values are in bold.

### Topic as alternative data

The universal model we built and described above does not use topics as features for training and inference. The reason is that we sought a model that can score any screening topic, not just the seven topics we have training data for. In Suppl. Table 5 we report how topics used as additional data for building a universal model reflect on the quality of the model. We can see that knowing topics helps the model to better score «confusing» IRD topic whereas other topic quality remains unchanged.

Before adding topic labels as alternative data, we balanced the dataset with regard to topics. This balancing resulted in cutting the drug abuse topic with DI labels fourfold. We observed in Suppl. Table 5 that this cut decreased the inferencing quality of the DA topic, which had always been an unexplained leader before. The quality of the DA topic became on par with a couple of the forerunning topics, such as corruption and criminal history. This observation explains the previous domination of the DA topic; it was because it benefited from a significantly larger minority class (DI label) than other topics.

### Ensembling and extra data

We measured the uplift from adding fresh 189 DIs and also experimented with various model ensembling architectures. The results are displayed in Table 8. Cumulatively, ensembling and extra data lifted AUC by 5% on all topics, and up to 11% on selected topics. Ensembling is explained in “Methods”.

**Table 8:** The impact of ensebling and extra data on AUC.

Universal model	Base model	Ensembling	+ 189 DIs	Ensembling and + 189 DIs
All topics	0.79 (0.01)	0.83 (0.01) ∆ = + 4%	0.84 (0.01) ∆ = ** + 5%**	0.84 (0.01) ∆ = ** + 5%**
Drug abuse	0.84 (0.02)	0.88 (0.01) ∆ = + 4%	0.84 (0.01) ∆ = 0%	0.88 (0.01)) ∆ = + 4%
Corruption	0.80 (0.03)	0.82 (0.04) ∆ = + 2%	0.88 (0.02) ∆ = ** + 8%**	0.86 (0.02) ∆ = ** + 6%**
Conf. info leak	0.72 (0.05)	0.79 (0.05) ∆ = ** + 7%**	0.85 (0.01) ∆ = ** + 13%**	0.83 (0.02) ∆ = ** + 11%**
Debt	0.75 (0.03)	0.80 (0.05) ∆ = ** + 5%**	0.84 (0.06) ∆ = ** + 9%**	0.82 (0.06) ∆ = ** + 7%**
Unrep. income	0.79 (0.07)	0.83 (0.05) ∆ = + 4%	0.85 (0.04) ∆ = ** + 6%**	0.83 (0.04) ∆ = + 4%
Criminal history	0.90 (0.01)	0.90 (0.01) ∆ = 0%	0.91 (0.04) ∆ = + 1%	0.91 (0.04) ∆ = + 1%
IRD violation	0.69 (0.02)	0.71 (0.03) ∆ = + 2%	0.75 (0.02) ∆ = ** + 6%**	0.73 (0.03) ∆ = + 4%

Significant values are in bold.

### Validating ML-based second-opinion in the field

We now have two advanced models: a Universal model (ensemble with alternative data), and a Drug Abuse model (one topic model with alternative data). Here we report the summary of the test to find examiner errors among 2094 field historical screenings. We selected screenings where the examiner concluded NDI, but a model voted for DI strongly.

Based on Drug Abuse model top scores, we selected 15 NDI examiner conclusions as candidates for examiner errors on drug abuse topic. Similarly, based on Universal model top scores, we selected 15/5/5 NDI conclusions on corruption/confidential information leak/criminal history topics. Thus, we ended up with 40 conclusions (candidates for examiner errors) in 36 screenings.

We handed these 36 screenings for thorough blind QA to two examiners. The examiners did not know the results of the screenings, and did not share their QA results with each other. The reason for performing two QAs is because if it happened that there was a discrepancy between the original conclusions and one QA, we would have the word of one examiner against the word of another examiner, which would not constitute an original examiner error per se. One screening (one conclusion on corruption topic) was later removed from QA procedure for technical reasons.

We have extremely experienced examiners, and there is a common assumption at the bank that the examiner error rate might be anywhere between 0.0 and 1.0% of all screenings. An examiner error is an extraordinary and critical situation that nobody remembers happening once during QAs performed from time to time for years. Thus, our test success criteria was to find at least one examiner error in 39 conclusions inside 35 screenings.

**Figure 2:**
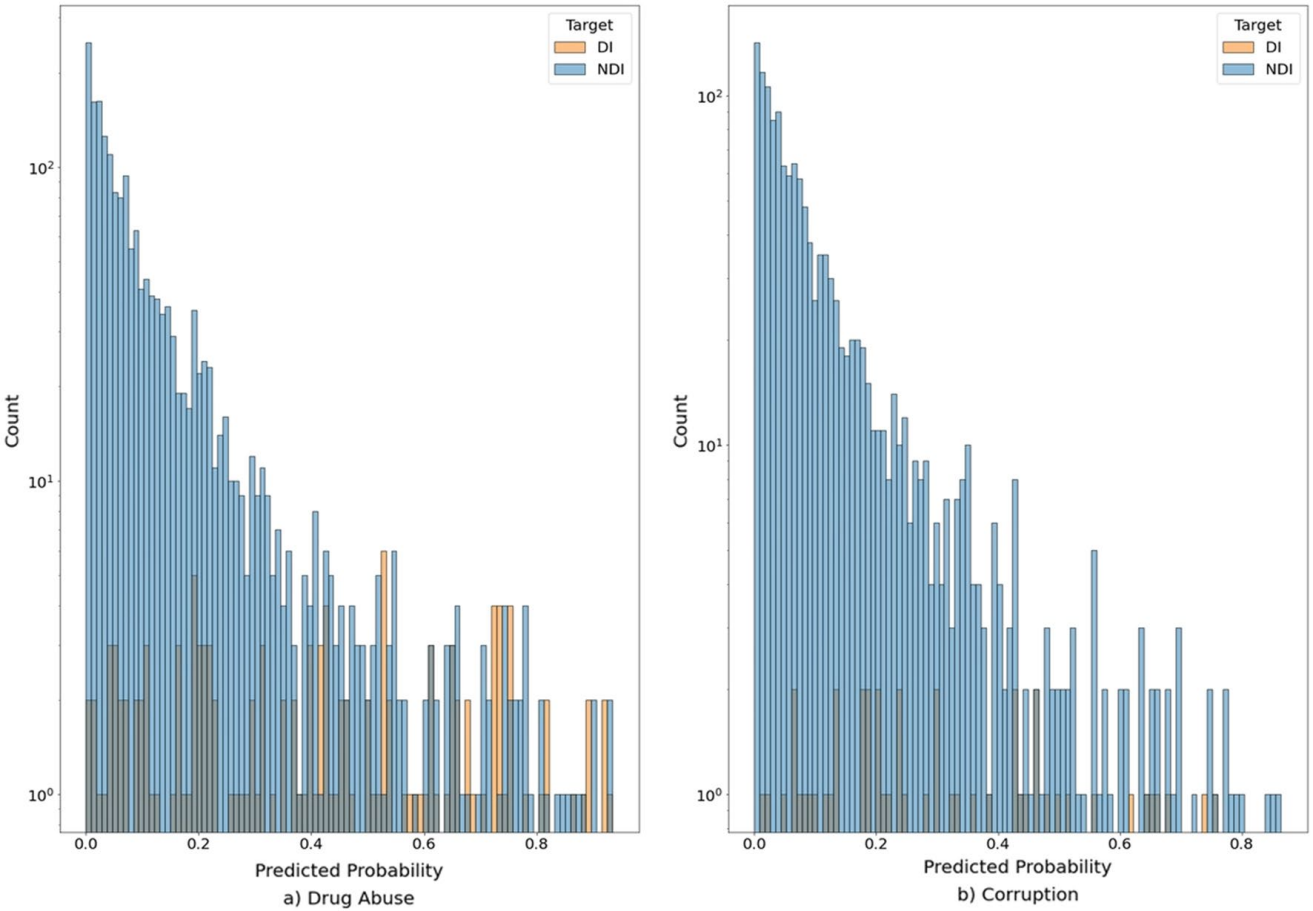
Distribution of basic model scores for topics: (**a**) “Drug abuse”; (**b**) “Corruption”.

The summary of the two QAs is presented in Table 9. The distributions of scores for two relevant topics are shown in Fig. 2.

**Table 9:** Results of two QAs of the top candidates for examiner error (EE) in 2094 screenings.

Screening	Drug abuse		Corruption		Conf. info leak		Crim. history		Total
	Score	Result	Score	Result	Score	Result	Score	Result	
1	0.959	ME	0.769	CN			0.840	ME	
2	0.933	CN							
3	0.932	CN							
4	0.931	EE							
5	0.903	CN			0.767	CN			
6	0.900	EE							
7	0.896	ME							
8	0.893	EE							
9	0.891	CN							
10	0.883	CN							
11	0.867	EE							
12	0.860	EE							
13	0.858	EE							
14	0.857	ME							
15	0.853	CN					0.838	ME	
16			0.888	CN					
17			0.826	CN					
18			0.804	EE					
19			0.798	CN					
20			0.787	CN					
21			0.765	ME					
22			0.739	EE					
23			0.734	ME					
24			0.732	EE					
25			0.729	CN					
26			0.729	ME					
27			0.728	EE					
28			0.727	EE					
29					0.817	CN			
30					0.803	EE			
31					0.769	CN			
32					0.767	CN			
33							0.813	EE	
34							0.808	CN	
35							0.799	ME	
Examiner errors		6		5		1		1	13
Concilium needed		6		6		4		1	17
Model errors		3		3		0		3	9

ME is model error (the suspicion in EE is disproven), CN is concilium needed (where QA1 and QA2 do not agree).

By QA-ing 39 examiner conclusions in 35 screenings (out of 2094 screenings) we identified 30 problematic conclusions, where either plain examiner errors are confirmed by two QAs (13 conclusions) or where QAs do not agree (17 conclusions). The remaining 9 conclusions are model errors, where an original examiner did not make a mistake as confirmed by both QAs. We expected that there would be some cases of QAs not concurring because some examiner mistakes could be hard calls where a decision is not obvious. For such hard cases, usually a concilium is called where examiners discuss their conflicting conclusions and come to an agreement. In this context, we are satisfied that a significant portion of the test (17 of 39 conclusions) has ended up in a concilium. It is dangerous to rely on hard call conclusions that require a concilium, without a concilium. We do not publish the results of the concilium since the results do not contribute towards the results of the paper.

We note that in *several* problematic screenings (DI set by one or both manual QAs), the examiners who conducted the QA made side notes that an examinee practiced counter-measures. Thus we conclude that our models catch some counter-measures. Missing a counter-measure is an examiner error by definition, but we were not sure we would catch anything beyond trivial errors.

We conclude that our models are fit for a one-year pilot, where 100% inflow of new screenings (approx. one hundred a day) will be scored. Manual QAs will be mandated in case of conflict between examiner conclusions and model scores on the topics. The exact model threshold will vary during the pilot, in part depending on the current load of the examiner team. The pilot will start at the end of 2022, after interfacing with a production polygraph report system is completed.

## Discussion

Slavcovic’s work on analyzing raw polygraph data twenty years ago remains the most relevant to our work. Similarities are that: (i) both studies work with raw classical polygraph data, (ii) both sets of data are collected in the field as opposed to data collected from volunteers instructed to lie, and (iii) accuracies of our models are roughly equivalent.

We differ in the following:

i. Slavcovic warned that data may contain examiner errors; we aimed at finding such errors and succeeded at catching examiner mistakes in historical records.
ii. We experimentally drew the lower bound of examiner error rate in the field (≈**1.5**%). This bound did not previously exist to the best of our knowledge. We believe this finding will provide factual motivation for QA.
iii. We showed the promise of novel data sources for accuracy of lie detection models, including examiner ID, examinee job role, wind, atmospheric pressure, and geomagnetic storms.
iv. We make part of the data accessible by reasonable request to facilitate academic research.
v. Data are of very different nature. Our data are hiring and regular screenings of civil personnel as opposed to Slavcovic's data from army criminal investigations.
vi. Distributions of classes (lie detected/not detected) differ significantly; our number of records is an order of magnitude higher; the classical polygraphs are of different manufacturers and decades; data sampling rates are 31 Hz vs 60 Hz (ours vs Slavkovic’s).
vii. Neither Slavkovic nor any work we know of looked into differentiating topics at training and at inferencing times. We showed that one can profit from both.

Differences v and vi make it hard to compare model accuracies; the nature of the data is very different. Even so, we were surprised that we did not achieve significantly better accuracy twenty years later. We agree with related work in that criminal investigations are easier to classify than routine civil personnel screenings^3^. Thus, we may have a significantly better model, but AUCs are on par with Slavcovic because emotions in our dataset are harder to classify, and because civil screening questions are much broader than criminal investigation questions.

Honts and Amato recruited 80 volunteers to mock lies and truthful answers in the screenings^29^. In half of the screenings, volunteers watched videotaped questions instead of an examiner asking questions, and a special algorithm (RI Score) scored the answers instead of an examiner evaluation. Honts and Amato conclude that the automated screening scenario was more accurate than that carried out by a human polygraph examiner. Honts and Amato neither aimed at finding, nor found, any examiner errors. We differ in that Honts and Amato automate the screenings while we automate examiner conclusion verification. We do not substitute examiner with automated scoring. Moreover, in our setup, we do not show the examiner the scores of our tool (to exclude the possibility of the tool results influencing the conclusion of the examiner). The RI Score is rule-based and apparently requires additional markup by an examiner to calculate, whereas our ML models need no markup in addition to NCCA ASCII standard.

Mambreyan et al. show that artificial bias in data with regards to sex leads to overestimating the quality of deception detection models running on video^30^. They refer to a work that built a model on a dataset of videos, where 65% of woman and only 27% of men lied. A model can learn sex from video, and use it to infer the truth/deception label, disregarding any other data. We made sure that we do not have artificial bias in the alternative data we use (sex, age, roles). Particulary for sex data, we demontstrate the balance in Suppl. Table 4.

Abouelenien et al. measure effectiveness of physiological, linguistic, and thermal features in deception detection on a laboratory dataset of size 149 and three synthetic topics (mock crime, attitude towards abortion, and best friend)^31^. We explore other alternative data sources, using field dataset and real topics, and in addition our main goal is a tool to hunt for examiner errors.

## Limitations

This is the first detailed disclosure of building and testing a second-opinion tool for classical polygraph. Yet, the subject is immense, and we may have just scratched the surface. To start with, the manual field validation (by double QA-ing candidates for examiner errors) covered only 39 conclusions but, as we explained, even this tiny test required approximately 80 examiner hours (not including a concilium to sort out discrepancies between the two QAs). We hope to grow these experimental statistics after putting the models to live pilot.

Our trainset is contaminated with few and unknown examiner errors and, at least until the field validation, we ran the risk of finding no examiner errors because of the models learning to make all the same mistakes examiners do. Making a gold standard trainset involves double QA-ing hundreds of screenings. While the field validation has proven that our second-opinion tool catches some examiner errors, we still cannot exclude the risk that models are confused by the most common examiner mistakes. The running of our tool in production slowly but surely will grow the golden dataset of screenings QA-ed by three examiners (and conclium in some cases), thus producing a first ever gold standard accessible to academics.

With manual QAs we validated errors where an examiner set NDI erroneously, but we did not investigate erroneous DI labels because we lack DI labels. Less than 7% of the screenings in the archive contain DI labels. Applying our work to detect this second type of errors is a direction for future work.

We decided to not implement examiner textbooks because in the examiner community we hear many discussions on exemptions to almost any textbook rule. To avoid being dragged into these heated, undocumented discussions we decided to use features that do not depend on examiner textbooks or scoring methods. We started with plain and simple raw signal features (min, mean, max on a window). We also started with gradient boosting models. The plan was to up our feature and model game after having the baseline research pipeline built. When we obtained 0.85 + AUC examiner conclusion inference quality, and keeping in mind that we shall avoid a perfect model as explained above, we decided that this is enough for a pilot. We believe that developing sophisticated raw signal features and employing neural networks more suited for time series (such as LSTM) is a good avenue for future work.

We tested several unorthodox data sources for uplift to conclusion prediction models. While there are some preliminary and promising results, most of these are inconclusive and need more data and investigation.

## Methods

### Ethics information

All methods were performed in accordance with relevant guidelines and regulations. This study neither required nor used any human participants. The study analyzes legacy polygraph screening data that is collected as part of a standard screening process of hiring candidates and employees with critical roles. The hiring candidates and employees sign a written agreement to be screened, including an informed consent for the Bank to store and to utilize the screening data. Internal Security of the Bank anonymized the data before handing it over to the authors of this study.

### Dataset description

We possess an archive of 2094 field polygraph screening recordings (PSRs) including Deception Indicated (DI) attributes set by examiners who conducted the screenings. These polygraph screenings (PS) were performed on bank personal with critical roles before hiring, before promotion, or every year, depending on their role. A PS includes a subset of 14 topics, including drug abuse and corruption.

PSRs store physiological signals of the examinee, audio, and questions as strings. Each question data includes three time-stamps relative to each repetition: the start of the question by the polygraph examiner (PE), the end of the question and the moment of the answer. Each question had a type assigned to it (see Suppl. Table 3 for the list of question types). In addition to physiological signals (listed in Suppl. Table 1) the examinee’s sex, age, and job position is recorded.

The screenings were performed on Polyconius polygraph, model 7.

### Feature engineering

The basic task was to make inference examiner conclusions (DI or NDI) for a certain topic in the screening.

To build a model we presented data in the following format: each row in the dataset is a record of PS by a certain topic in a particular test for an examinee. Targets are DI or NDI. There may be a bias due to such target-setting, because an examinee may not lie in all tests on a topic during screening.

Physiological signals were extracted from a time window defined by time-stamps of the question’s data. Thus, initially a row is a time series of the physiological signals for a given repetition on given topic.

For every repetition of the relevant and comparison questions we generate the basic statistics: minimum, maximum, mean, amplitude and standard deviation. Further, we used minimum, maximum, mean and standard deviation as aggregate functions at each step. The repetition’s data we grouped by the question. An additional feature is to characterize the difference between the first repetition and the next ones. Similarly, the question’s data we grouped by topic for each test. At the end, each row in the dataset comprises 600 features extracted from PSR for a certain topic in a particular test (Suppl. Fig. 1) for an examinee, with the label (DI/NDI).

### Models

We used gradient boosting with a two-level stacking ensemble to avoid the curse of dimensionality. The first-level model trained on 600 physiological features for a topic, inferencing DI/NDI for each test inside a screening. The second-level model aggregated the output of the first-level model for all tests for a topic. At the second-level we have the following features:

- pred_proba_max—the maximal probability of DI among the tests;
- pred_proba_mean—the mean probability of DI;
- pred_proba_min—the minimal probability of DI among the tests;
- pred_proba_diff—difference between the maximal and mean values.

These probabilities are concatenated with alternative data (biographical data, weather data, geomagnetic storm data). The obtained dataset is fed to the input of the second model, which gives the probability of DI on screening for a topic.

### Basic model

**Figure 3:**
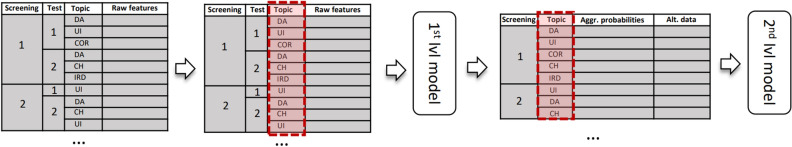
Universal model scheme. Information that is not received by the model is marked red.

This model does not receive information about topics during training and inference (Fig. 3). Information about topic type is saved for further aggregation by screening. For example, the drug abuse (DA) topics of each test are aggregated in the first screening. The second-level model result is an estimation of probability of DI for any given topic of a screening.

### One-topic model

**Figure 4:**
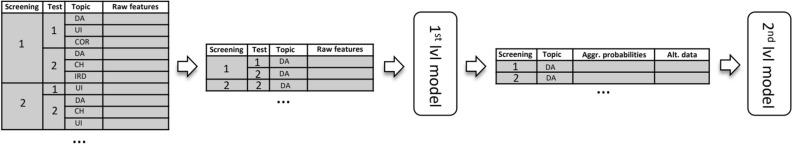
One-topic model scheme.

The logic of model construction and feature generation is the same as in the basic model. The difference is that for training, we used features of one topic only. The data is filtered by a single topic before the first-level model is applied. Figure 4 shows how the ensemble is trained on the drug abuse topic, while other topics are filtered out.

### Universal model

**Figure 5:**
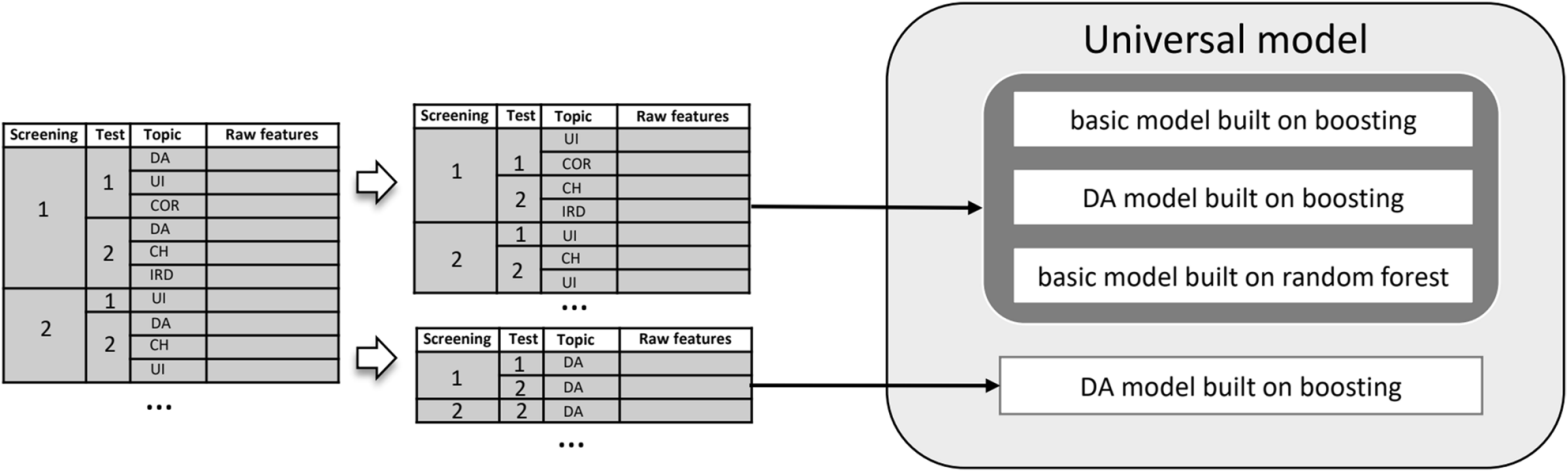
Universal model scheme.

We decided to use the best sides of the models described above, so we built an ensemble of existing architectures. After a series of experiments, the architecture showed the best result where we use averaging the confidence of the following models (Fig. 5):

- a basic model built on boosting, using alternative data;
- a model of a single topic, built on boosting, using alternative data;
- a basic model built on a random forest.

This ensemble was applied to all topics except drug abuse. Aside from traditional advantages of ensembles, here the rationale for using models of different architectures (e.g. gradient boosting and random forest) together is that it hopefully will eliminate some pure model errors and will highlight label (target) errors that constitute examiner errors.

### Training

Since we did not have much data (2094 files), we used the stratified group K-Fold Validation to evaluate each of the historical screening. We set the K value equal to 5 and, using the developed a framework of stacking standard models, and received 5 models, trained on 80% of data each.

The custom values that we used as hyperparameters for the standard classifiers of the open source libraries are presented in Table 10.

**Table 10:** Hyperparameters of standard models used in the framework.

Classifier (library)	Hyperparameter	Value
Gradient boosting (CatBoost^32^)	auto_class_weights	Balanced
	cat_features	None (for 1st lvl model)
		list of feature indexes: sex, job position data (for 2nd lvl model)
Random forest (scikit-learn^33^)	max_depth	5
	class_weight	Balanced

Standard hyperparameters can be found in the documentation of the open source ML frameworks, links to the documentation are in Suppl. Table 7.

### Validating

We evaluated the quality of the model using a test set of each validation step described in the Training subsection above. Thus, at this stage, we evaluate success of the model in the classical understanding of machine learning, i.e. as improvements in the main metrics (ROC-AUC, TPR, FPR).

### Testing

Our main focus was not to build a high quality ML model for polygram classification, but to use an ML model to detect I-type ML model errors (FP) in polygraph screenings. Since a I-type error from the model’s perspective is equivalent to the II-type error (FN) from an examiner’s point of view, this procedure allowed us to find potential labeling errors in our historical sample, where an examiner did not indicate deception when the deception should have been indicated. After we got desired results at the validation stage, we use an expensive resource, examiners, to re–check polygrams that were likely to contain examiners’ errors, as explained above in the “Results” section, “Validating ML-based second-opinion in the field” section.

## Supplementary Information

Supplementary Information.

## Data Availability

Upon reasonable request we are ready to provide part of our anonymized screening dataset, subject to a non-exclusive, revocable, non-transferable, and limited right to use the data for the exclusive purpose of undertaking academic research. However, in 2022 a federal law was signed that complicates cross-border personal data transfer^34^. Anonymized datasets are deemed personal data too. In case of our anonymized data, a lengthy approval process will be required for each recipient, without any success guarantees. We devised a way to offer academic researchers the opportunity to experiment with the anonymized dataset, so that no data is transferred cross-border. Upon reasonable request, we will accept Python scripts, run those against the dataset, and report back the quality metrics of the resulting model. To ease experiment preparation, we are ready to share detailed data structure description and baseline code. The data is in NCCA ASCII standard text format^35^, and has a 31 Hz sampling rate. It contains 2094 anonymized field screenings, including date, raw signal data, alternative data (sex and age), magnetic storm data, weather data for one city, labels for seven topics (DI/NDI), and questions and answers time-stamped and labeled for seven topics.

## References

[1] Harris, M. The lie generator: inside the black mirror world of polygraph job screenings. Wired, https://www.wired.com/story/inside-polygraph-job-screening-black-mirror/ (2018).

[2] Banerjee, B. & Chatterjee, G. The world of lie detection: a study into state of lie detection usage by state and society in Asia, Africa and Europe. Preprint at https://osf.io/preprints/socarxiv/8hj69/ (2021).

[3] National Research Council (2003). The Polygraph and Lie Detection.

[4] Slavkovic, A. Evaluating polygraph data. https://www.stat.cmu.edu/tr/tr766/tr766.pdf (Carnegie Mellon University, 2002).

[5] Synnott J, Dietzel D, Ioannou M (2015). A review of the polygraph: History, methodology and current status. Crime Psychol. Rev..

[6] Krishnamurthy, G., Majumder, N., Poria, S. & Cambria, E. A deep learning approach for multimodal deception detection. In *19th Int. Conference on Computational Linguistics and Intelligent Text Processing (CICLing)* (2018).

[7] Avola, D., Cinque, L., Foresti, G. L. & Pannone, D. Automatic deception detection in RGB videos using facial action units. In *13th Int. Conference on Distributed Smart Cameras (ICDSC)* (2019).

[8] Samadiani N (2019). A review on automatic facial expression recognition systems assisted by multimodal sensor data. Sensors.

[9] Webb AK, Honts CR, Kircher JC, Bernhardt P, Cook AE (2010). Effectiveness of pupil diameter in a probable-lie comparison question test for deception. Legal Criminol. Psychol..

[10] Walczyk JJ, Mahoney KT, Doverspike D, Griffith-Ross DA (2009). Cognitive lie detection: Response time and consistency of answers as cues to deception. J. Bus. Psychol..

[11] Shuster A (2021). Lie to my face: An electromyography approach to the study of deceptive behavior. Brain Behav..

[12] Abootalebi V, Moradi MH, Khalilzadeh MA (2009). A new approach for EEG feature extraction in P300-based lie detection. Comput. Methods Prog. Biomed..

[13] Kozel A (2005). Detecting deception using functional magnetic resonance imaging. Biol. Psychiatry.

[14] Farah MJ, Hutchinson JB, Phelps EA, Wagner AD (2014). Functional MRI-based lie detection: Scientific and societal challenges. Nat. Rev. Neurosci..

[15] Monaro M (2018). Covert lie detection using keyboard dynamics. Sci. Rep..

[16] Sousedikova L, Hromada M, Adamek M (2021). Analysis of Artificial Intelligence Lie Detector Developed for Airport Security.

[17] Sánchez-Monedero J, Dencik L (2022). The politics of deceptive borders: biomarkers of deceit and the case of iBorderCtrl. Inf. Commun. Soc..

[18] Quijano-Sánchez L, Liberatore F, Camacho-Collados J, Camacho-Collados M (2018). Applying automatic text-based detection of deceptive language to police reports: Extracting behavioral patterns from a multi-step classification model to understand how we lie to the police. Knowl.-Based Syst..

[19] Editorial. Police use a computer to expose false testimony. *Nature* **557** (2018). 10.1038/d41586-018-05285-9 29849162

[20] Ben-Shakhar G, Iacono W (2021). Fallacies in the estimation of the validity of the Comparison Question Polygraph Test: A reply to Ginton (2020). Investig. Psychol. Offender Profiling.

[21] Grubin D, Madsen L (2005). Lie detection and the polygraph: A historical review. J. Forensic Psychiatry Psychol..

[22] Hinkle C (2021). The modern lie detector: AI-powered affect screening and the Employee Polygraph Protection Act (EPPA). Georgetown Law J..

[23] Bittle J (2020). Lie Detectors have Always Been Suspect. AI has made the Problem Worse.

[24] Saxe L (1991). Science and the CQT polygraph—A theoretical critique. Integr. Physiol. Behav. Sci..

[25] Perkey AM (2021). Recommendations for Uniform Polygraph Examinations for Preemployment Screening of Law Enforcement Applicants.

[26] Egerton W (2020). Use of the Polygraph to Screen Police Candidates.

[27] Baur, D. Federal Psychophysiological Detection of Deception Examiner Handbook. *Counterintelligence Field Activity Technical Manual* (2006).

[28] Matzka, J., Bronkalla, O., Tornow, K., Elger, K. & Stolle, C. Geomagnetic Kp index V. 1.0., *GFZ Data Services.*10.5880/Kp.0001 (2021).

[29] Honts CR, Amato S (2007). Automation of a screening polygraph test increases accuracy. Psychol. Crime Law..

[30] Mambreyan, A., Punskaya, E. & Gunes, H. Dataset bias in deception detection. In *26th Int. Conference on Pattern Recognition (ICPR)* (2022).

[31] Abouelenien M, Pérez-Rosas V, Mihalcea R, Burzo M (2016). Detecting deceptive behavior via integration of discriminative features from multiple modalities. IEEE Trans. Inf. Forensics Security.

[32] Prokhorenkova, L., Gusev, G., Vorobev, A., Dorogush, A. V. & Gulin, A. CatBoost: Unbiased boosting with categorical features. *Adv. Neural Inf. Process. Syst.* **31**, 6639–6649 (2018).

[33] Pedregosa F, Varoquaux G, Gramfort A, Michel V, Thirion B, Grisel O (2011). Scikit-learn: Machine learning in Python. J. Mach. Learn. Res..

[34] Interfax, Bill on possible ban on transfer abroad of Russians' personal data being submitted to State Duma https://interfax.com/newsroom/top-stories/77833/ (2022).

[35] Handler, M. & Hernandez, N., Introduction to the NCCA ASCII standard. *Polygraph Forensic Credibil. Assess. J. Sci. Field Pract.* **48**(2), 125–135 (2019).

