# Supplementary material for: Building a second-opinion tool for classical polygraph
Source: Sci Rep. 2023 Apr 17;13:5522. doi: 10.1038/s41598-023-31775-6 (PMC10110587; doi:10.1038/s41598-023-31775-6)
Supplement: Supplementary file 1 — Supplementary Information. [file 41598_2023_31775_MOESM1_ESM.pdf]

Building a Second-Opinion Tool for Classical Polygraph

Dmitri Asonov[1], Maksim Krylov[23], Vladimir Omelyusik[1], Anastasiya Ryabikina[2],
Evgeny Litvinov[1], Maksim Mitrofanov[2], Maksim Mikhailov[2], Albert Efimov[1,4]

Supplementary material

Supplementary Table 1 (Polygraph raw data description):

Raw signal	Description
ABDOMINAL_RESP	Abdominal respiration
ABS_BLOOD_VOLUME	Absolute blood volume
BLOOD_VOLUME	Relative blood volume
EDA	Electrodermal activity
HEART_RATE	Heart rate
OPTIONAL	Additional sensor of examiner choice
PLE	Photoelectric plethysmograph
THORACIC_RESP	Thoracic respiration
TONIC_EDA	Tonic electrodermal activity
TREMOR	Tremor

Supplementary Table 2 (Screening topics description):

Screening topic (risk factor)	Description (sample question)
Drug abuse	*Have you consumed illegal drugs in the previous 12 months?*
Corruption	*Have you received illegal remuneration from a client ever?*
Conf. info leak	*Have you used information containing bank secrecy for personal purposes?*
Debt	*Are you currently experiencing difficulties dealing with debt?*
Unrep. income	*Do you receive income from outside of the corporation that you have not reported?*
Criminal history	*Have you ever been found guilty of a criminal offense by Court?*
IRD violation	*Have you ever violated an Internal (Corporate) Regulatory Document?*

Supplementary Table 3 (Question types):

Question type / in Russian	Description
Relevant / Пв	Questions directly related to the focus of an investigation
Comparison / Вс	Questions for which the responses are designed to be compared to responses to relevant questions
Irrelevant / Нв	Questions which are believed to have no, or very little, emotional impact on a subject
Sacrifice relevant / 0, Жв	Question which makes it possible to reduce the probability of error, which is said to be the result of evaluating the first relevant question in the series

Supplementary Table 4 (Testing for bias in sex attribute in the dataset):

Sex	All topics		Drug abuse		Corruption		IRD violation	
	Count	% of DI	Count	% of DI	Count	% of DI	Count	% of DI
Male	1222	13,7%	1181	7,9%	817	4%	724	2,8%
Female	872	10,7%	831	5,3%	619	2,4%	705	3,7%

[1] Sberbank of Russia, Sber Innovation and Research, Moscow, Russian Federation
[2] Sberbank of Russia, Internal Security Department, Moscow, Russian Federation,
[3] Contact author: MAKrylov@sberbank.ru
[4] NUST MISIS

Supplementary Figure 1 (Convolution of raw data)

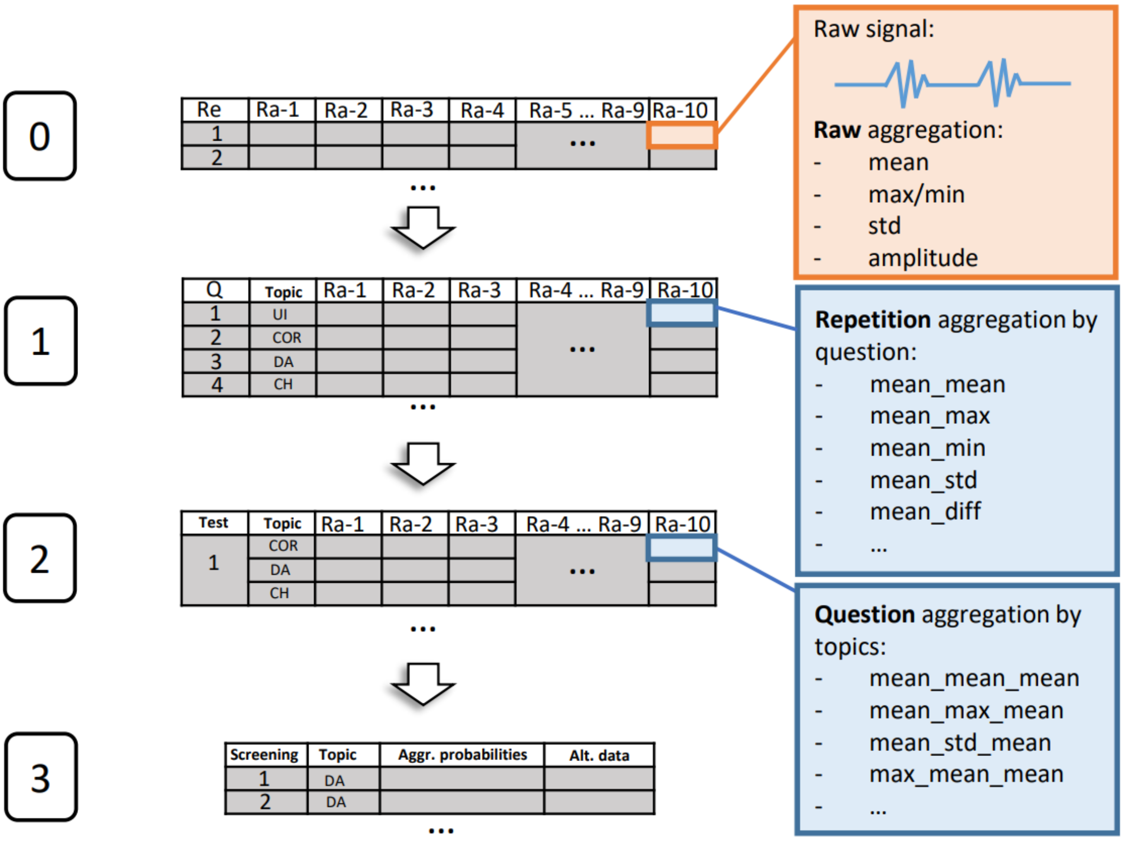

Supplementary Table 5 (Three Models built on cut but balanced by DIs dataset: a) One topic model; b) Basic model without feature "topic"; c) Basic model with feature "topic"):

	All topics	Drug abuse	Corruption	Conf. info leak	Debt	Unrep. income	Crime history	IRD violation
ROC AUC (Std.)	a. 0.67 (0.02)<br>b. 0.67 (0.02)<br>c. 0.68 (0.02)	0.80 (0.04)<br>0.67 (0.10)<br>0.69 (0.05)	0.66 (0.04)<br>0.68 (0.04)<br>0.66 (0.06)	0.70 (0.04)<br>0.67 (0.04)<br>0.71 (0.03)	0.66 (0.06)<br>0.65 (0.04)<br>0.64 (0.03)	0.67 (0.03)<br>0.67 (0.06)<br>0.67 (0.04)	0.65 (0.03)<br>0.74 (0.05)<br>0.70 (0.04)	0.53 (0.04)<br>0.65 (0.06)<br>0.66 (0.03)
Recall (Std.)	a. 0.12 (0.01)<br>b. 0.16 (0.03)<br>c. 0.17 (0.01)	0.21 (0.08)<br>0.19 (0.10)<br>0.10 (0.06)	0.08 (0.04)<br>0.12 (0.03)<br>0.09 (0.06)	0.21 (0.1)<br>0.09 (0.03)<br>0.19 (0.05)	0.09 (0.06)<br>0.18 (0.12)<br>0.12 (0.05)	0.07 (0.04)<br>0.16 (0.04)<br>0.05 (0.02)	0.07 (0.07)<br>0.15 (0.09)<br>0.15 (0.09)	0.10 (0.04)<br>0.23 (0.07)<br>0.27 (0.04)
Precision (Std.)	a. 0.04 (0.00)<br>b. 0.06 (0.01)<br>c. 0.06 (0.00)	0.09 (0.04)<br>0.05 (0.03)<br>0.15 (0.09)	0.04 (0.02)<br>0.06 (0.02)<br>0.06 (0.04)	0.05 (0.02)<br>0.04 (0.02)<br>0.07 (0.03)	0.02 (0.02)<br>0.05 (0.02)<br>0.06 (0.03)	0.03 (0.02)<br>0.07 (0.02)<br>0.02 (0.02)	0.01 (0.01)<br>0.05 (0.04)<br>0.05 (0.04)	0.02 (0.01)<br>0.08 (0.02)<br>0.08 (0.01)
F1_score (Std.)	a. 0.06 (0.00)<br>b. 0.08 (0.01)<br>c. 0.09 (0.01)	0.10 (0.04)<br>0.08 (0.04)<br>0.12 (0.07)	0.06 (0.02)<br>0.07 (0.02)<br>0.07 (0.05)	0.08 (0.03)<br>0.05 (0.02)<br>0.10 (0.01)	0.04 (0.02)<br>0.07 (0.03)<br>0.08 (0.03)	0.04 (0.03)<br>0.10 (0.02)<br>0.02 (0.02)	0.02 (0.02)<br>0.07 (0.05)<br>0.07 (0.06)	0.04 (0.02)<br>0.12 (0.03)<br>0.13 (0.02)
Accuracy (Std.)	a. 0.93 (0.01)<br>b. 0.94 (0.00)<br>c. 0.94 (0.00)	0.94 (0.01)<br>0.94 (0.00)<br>0.96 (0.01)	0.93 (0.01)<br>0.93 (0.01)<br>0.92 (0.01)	0.94 (0.01)<br>0.94 (0.01)<br>0.94 (0.01)	0.94 (0.01)<br>0.93 (0.00)<br>0.92 (0.00)	0.94 (0.01)<br>0.95 (0.00)<br>0.95 (0.01)	0.93 (0.01)<br>0.94 (0.00)<br>0.93 (0.00)	0.91 (0.01)<br>0.93 (0.01)<br>0.92 (0.01)
TNR (Std.)	a. 0.95 (0.01)<br>b. 0.95 (0.00)<br>c. 0.95 (0.00)	0.95 (0.01)<br>0.96 (0.00)<br>0.97 (0.01)	0.94 (0.01)<br>0.95 (0.01)<br>0.94 (0.01)	0.95 (0.01)<br>0.95 (0.01)<br>0.95 (0.01)	0.96 (0.01)<br>0.95 (0.01)<br>0.93 (0.01)	0.96 (0.00)<br>0.96 (0.00)<br>0.96 (0.01)	0.95 (0.01)<br>0.95 (0.01)<br>0.94 (0.01)	0.92 (0.01)<br>0.94 (0.01)<br>0.93 (0.01)
FPR (Std.)	a. 0.05 (0.01)<br>b. 0.05 (0.00)<br>c. 0.05 (0.00)	0.05 (0.01)<br>0.04 (0.00)<br>0.03 (0.01)	0.06 (0.01)<br>0.05 (0.01)<br>0.05 (0.01)	0.05 (0.01)<br>0.05 (0.01)<br>0.05 (0.01)	0.04 (0.01)<br>0.05 (0.01)<br>0.07 (0.01)	0.04 (0.00)<br>0.04 (0.00)<br>0.04 (0.01)	0.05 (0.01)<br>0.05 (0.01)<br>0.06 (0.01)	0.08 (0.01)<br>0.06 (0.01)<br>0.07 (0.01)
Number of DI	158	30	30	30	30	24	24	30

Supplementary Table 6 (Description of alternative data):

	**Feature**	**Description**
1	subject_age	Age of the examinee
2	pred_proba_min	The minimal probability of DI among the tests (more details in methods)
3	pred_proba_max	the maximal probability of DI among the tests (more details in methods)
4	examiner_id	Examiner ID
5	pred_proba_mean	the mean probability of DI (more details in methods)
6	Pressure	Atmospheric pressure on a given date
7	current_position	Current work position of the examinee
8	Wind	Wind direction on a given date
9	Dew Point	Dew point on a given date
10	accepted_position	Accepted work position of the examinee
11	Humidity	Humidity on a given date
12	pred_proba_diff	Difference between the maximal and mean values (more details in methods)
13	current_department	Current department of the examinee
14	Condition	Weather condition on a given date (rainy, windy, snowy etc.)
15	Temperature	Temperature on a given date
16	Time	Time of the examination
17	accepted_department	Accepted department of the examinee
18	Wind speed	Wind speed on a given date
19	subject_sex	Gender of the examinee
20	subject_type	Examinee type (candidate, employee etc.)

Supplementary Table 7 (Documentation of open source classifiers with all standard hyperparameter values)

**Classifier**	**Link to documentation with default hyperparameters**
Gradient boosting (CatBoost)	https://catboost.ai/en/docs/concepts/python-reference_catboostclassifier
Random Forest (Scikit-learn)	https://scikit-learn.org/stable/modules/generated/sklearn.ensemble.RandomForestClassifier.html
